# On the Interval Stability of Weak-Nonlinear Control Systems with Aftereffect

**DOI:** 10.1155/2016/6490826

**Published:** 2016-10-23

**Authors:** Andriy Shatyrko, Denys Khusainov

**Affiliations:** Department of Complex Systems Modelling, Cybernetics Faculty, Taras Shevchenko National University of Kyiv, Volodymyrska Str., 64, Kyiv 01601, Ukraine

## Abstract

Sufficient conditions of interval absolute stability of nonlinear control systems described in terms of systems of the ordinary differential equations with delay argument and also neutral type are obtained. The Lyapunov-Krasovskii functional method in the form of the sum of a quadratic component and integrals from nonlinearity is used at construction of statements.

## 1. Introduction

The actuality of absolute interval stability problem of the dynamical systems, mentioned in the present paper, proves to be true as a lot of interesting reports at the international congresses and conferences and set of foreign publications, for example, [1–7]. The paper [7] is very interesting, because it gives us the most extensive, of the hitherto known, list of citations (825 point till 2006 year).

The investigation problems of dynamic systems with inexact specified parameters, or more, with the velocity vector (right-hand sides of differential equations), which can take their values from some of the sets, interested researchers for a long time. Classical (Lyapunov) stability means investigation of solutions at indignations by the initial data [8]. Its various generalizations (uniform on time and phase variables, by parts variables, asymptotical, exponential, orbital, etc.) also meant the unequivocal set of the law of dynamics of systems.

The solution of practical problems of control theory has caused occurrence concept “robust” (or interval) stability. Originally under robust stability, asymptotical stability of the linear stationary differential equations of the higher order was understood, under condition of a finding of their coefficients in the set intervals beforehand. Interesting fundamental necessary and sufficient conditions of interval stability of the linear differential equations with uncertainty parameters have been obtained at papers of Kharitonov [9–12]. However, at distribution of the obtained results to the dynamical systems, on differences equations and systems of the equations, systems with aftereffect have arisen essential difficulties.

The solution of control problems in linear systems leads to a finding of function (scalar function) *u*(*x*), at which feedback system

(1) $$\begin{array}{c} \dot{x}\left(\;t\;\right)=Ax\left(\;t\;\right)+bu\left(\;x\left(\;t\;\right)\;\right) \end{array}$$

should be asymptotically stable. Often this function depends on one scalar argument representing a linear combination of phase coordinates and some scalar function from the first and third squares of a plane. Investigations of asymptotical stability of the systems

(2) $$\begin{array}{c} u\left(\;x\left(\;t\;\right)\;\right)=f\left(\;\sigma \left(\;t\;\right)\;\right),\;\sigma \left(t\right)=c^{T}x\left(t\right), \end{array}$$

that is, systems

(3) $$\begin{array}{c} \dot{x}\left(\;t\;\right)=Ax\left(\;t\;\right)+bf\left(\;\sigma \left(\;t\;\right)\;\right),\;\sigma \left(t\right)=c^{T}x\left(t\right),\;t\geq 0, \end{array}$$

with continuous function *f*(*σ*), lying in the set sector, became known as the absolute stability investigations of regulating (or control) systems.

**Definition 1 (see [13, 14]).**

The nonlinear control system (3) is called absolutely stable if its trivial solution is globally asymptotically stable for arbitrary function *f*(*σ*), which belongs to the given linear sector:

(4) $$\begin{array}{c} 0<f\left(\;\sigma \;\right)\sigma \leq k\sigma^{2},\;k=const>0,\;f\left(0\right)=0. \end{array}$$

Problems of control systems absolute stability have arisen in the middle of last century and are connected with problems of stabilization of programmed control at the set structure of control function [6, 13, 14]. The results giving absolute stability conditions, that is, stability as a whole the zero solution for the set class of nonlinearity, have been obtained in two directions.

One approach of investigations here is the so-called “frequency method” that had development in Yakubovich et al. works [15–18]. At the heart of a method is a study of behavior of some curve (“godograph”) which lies in complex area. This approach is well developed on case of system with delay argument in works of Romanian scientist Rasvan [19].

Another alternative approach which has had development in works by Barbashin et al. is the Lyapunov second (direct) method with function type of “quadratic form plus integral from nonlinearity” [20–23].

Distribution of this method on systems with delay and neutral type has been obtained in Shatyrko and Khusainov works [24–27] and numerous works of Chinese scientists (e.g., [5, 28]). Sufficient conditions of absolute interval stability have been constructed. At their construction the finite-dimensional method of Lyapunov's functions with a condition of Razumikhin [29] was used. The condition of Razumikhin facilitates solving the investigation problem of sign-definiteness of Lyapunov function total derivative along the system solution. By means of this approach it is possible to estimate influence of aftereffect, that is, to obtain the conditions of absolute interval stability depending on delay. However, the conditions of Razumikhin impose rigid enough restrictions on aftereffect. And their use is not always effective.

At this paper we will use an alternative method of Lyapunov-Krasovskii functionals [3, 5, 30, 31]. The most effective functionals are the integrated additives of a quadratic type. At this approach the obtained estimations become simpler. However, here as a point of phase space all pieces of a trajectory are considered; therefore, the approach does not allow us to estimate influence of delay on absolute stability. Besides, the total derivative represents the quadratic form from phase coordinate and its prehistory. Therefore, the matrix of the quadratic form of a total derivative has twice the big dimension.

## 2. Direct Control Systems with Time-Delay Argument

At this section we will consider the system of direct control described by the differential equations with interval coefficients and with delay argument of the next type:

(5) x˙t=A+ΔAxt+B+ΔBxt−τ+bfσt,σt=cTxt.

In (5) *A*, *B*, Δ*A*, and Δ*B* are *n* × *n* constant matrices, *b*, *c* are constant *n*-vector column, and *τ* > 0 is constant delay. Together with system (5) consider initial condition

(6) $$\begin{array}{c} x\left(\;t\;\right)=\varphi \left(\;t\;\right), \end{array}$$

where *φ* : [−*τ*, 0] → *ℜ* ^*n*^ is an arbitrary continuously differentiable function.

Elements of matrices Δ*A* and Δ*B* also accept values from the fixed intervals:

(7) ΔA=Δaij,Δaij≤αij,  i,j=1,n¯,ΔB=Δbij,Δbij≤βij,  i,j=1,n¯.

Nonlinear function *f*(*σ*) satisfies the “sector condition” (4).

**Definition 2 .**

System (5) is called (Δ*A*, Δ*B*) interval absolutely stable, if it is absolutely stable for arbitrary matrices Δ*A* and Δ*B* from given intervals (7).

Under stability, asymptotic stability, and global stability of the delay system solution we understand traditional definitions; for example, see [31].

At Shatyrko and Khusainov earlier papers conditions of interval stability of systems (5) using finite-dimensional Lyapunov's functions

(8) $$\begin{array}{c} V\left(\;x\;\right)=x^{T}Hx+\beta \overset{\sigma \left(x\right)}{\underset{0}{\int }}f\left(\;\xi \;\right)d\xi ,\;\sigma \left(x\right)=c^{T}x \end{array}$$

have been obtained [24–27].

At the present paper we will construct conditions of interval stability of system (5) with the help of Lyapunov-Krasovskii functional

(9) Vxt=xTtHxt+∫−τ0xTt+sGxt+sds+β∫0σtfξdξ,σt=cTxt.

Throughout the paper we will use the following notation.

Let *S* be a real symmetric square matrix. Then the symbol *λ*min⁡(*S*)  (*λ*max⁡(*S*)) will denote the minimal (maximal) eigenvalue of *S*. We will also use the following vector norms:

 |*x*(*t*)| = {∑_*i*=1_^*n*^ *x*_*i*_^2^(*t*)}^1/2^: vectors norm (the Euclidean norm) in *C*_0_ space.
 ‖*x*(*t*)‖_2_ = {∫_−*τ*_^0^|*x*(*t* + *s*)|^2^ *ds*}^1/2^: vectors norm in *L*_2_ space.

For the real matrices we will use correspondent singular norm |*A*| = {*λ*_max_(*A* ^*T*^ *A*)}^1/2^, and next notations ‖Δ*A*‖ = max_Δ*a*_*ij*__⁡{|Δ*A*|}⁡; ‖Δ*B*‖ = max_Δ*b*_*ij*__⁡{|Δ*B*|}.

*θ* is the zero-vector; Θ is the zero-matrix, and *I* is the identity diagonal matrix.

Let us preliminary consider delay system without “interval perturbations”:

(10) x˙t=Axt+Bxt−τ+bfσt,σt=cTxt.

**Theorem 3 .**

Let the positive definite matrices *G*, *H* exist and parameter *β* > 0 such that the matrix

(11) SG,H,β=−ATH−HA−G−HB−Hb+12βAT+Ic−BTHGθ−Hb+12βAT+IcTθT1k−βbTc

is positive definite too. Then system (10) with delay without interval perturbations is absolutely stable.

**Proof**

As function *f*(*σ*) satisfies condition (4), then for functional (9) the following bilateral estimations are true:

(12) λminHxt2+λminGxt22≤Vxt≤λmaxH+kβc2xt2+λmaxGxt22.

We will calculate a total derivative of functional along system solutions. We will obtain

(13) ddtVxt=Axt+Bxt−τ+bfσtTHxt+xTtHAxt+Bxt−τ+bfσt+xTtGxt−xTt−τGxt−τ+βfσtcTAxt+Bxt−τ+bfσt.

Or, using the so-called *S*-procedure [17],

(14) ddtVxt−xTt,xTt−τ,fσt·SG,H,β·xTt,xTt−τ,fσtT.

If the matrix *S*[*G*, *H*, *β*] is positive definite, then

(15) ddtVxt≤−λminSG,H,β·xt2+xt−τ2+fσt2.

Thus, according to Krasovskii weak theorem [31] if there exist the positive definite matrices *G*, *H* and *S*[*G*, *H*, *β*], such that

(16) λminHxt2Vxt≤λmaxH+kβc2xt2+λmaxGxt22,ddtVxt−λminSG,H,βxt2,

then delay system (10) is absolutely stable.

Further we will obtain conditions of absolute interval stability of system (5).

**Theorem 4 .**

Let the positive definite matrices *G*, *H* exist and parameter *β* > 0, such that the next inequality is true

(17) λminSG,H,β>ΔA×H+ΔA2H2+ΔB2H2+14β2ΔA2c2.

Then system (5) is (Δ*A*, Δ*B*) interval absolutely stable.

**Proof**

As appears from the type of functional (9), bilateral estimations (12) are fair. We will calculate a total derivative of functional along solutions of system with “interval perturbations.” We will obtain

(18) ddtVxt−xTt,xTt−τ,fσt·SG,H,β·xTt,xTt−τ,fσtT+xTt,xTt−τ,fσt·ΔSG,H,β·xTt,xTt−τ,fσtT,

where

(19) $$\begin{array}{c} \Delta S\left[\;G,H,\beta \;\right]=\left[\begin{array}{ccc} \Delta A^{T}H+H\Delta A & H\Delta B & \frac{1}{2}\beta \Delta A^{T}c\\ \Delta B^{T}H & \Theta & \theta \\ \frac{1}{2}\beta c^{T}\Delta A & \theta^{T} & 0 \end{array}\right]. \end{array}$$

If the matrix *S*[*G*, *H*, *β*] is positive definite, then

(20) ddtVxt≤−λminSG,H,β·xt2+xt−τ2+fσt2+2ΔA×H×xt2+2ΔB×H×xt×xt−τ+βΔA×c×xt×fσt.

From here we have

(21) ddtVxt≤−λminSG,H,β−2ΔAHxt2−λminSG,H,βxt−τ2−λminSG,H,βfσt2+2ΔB×H×xt×xt−τ+βΔA×c×xt×fσt.

Let us rewrite the first term from right side of inequality in two parts and we will present (21) as follows:

(22) ddtVxt≤−αλminSG,H,β−2ΔA×Hxt2−2ΔB×H×xt×xt−τ+λminSG,H,β×xt−τ2−1−α·λminSG,H,β−2ΔA×Hxt2−βΔA×c×xt×fσt+λminSG,H,βfσt2,

where 0 < *α* < 1, some constant. Then, as appears from Sylvester's criterion [32], performance of inequalities will be a condition of absolute interval stability of system with delay:

(23) λminSG,H,β−2ΔA×H>0,αλminSG,H,β−2ΔA×H·λminSG,H,β−ΔB×H2>0,1−αλminSG,H,β−2ΔA×H·λminSG,H,β−14βΔA×c>0.

Let Δ*A* be such that the first inequality is executed. We will rewrite the second and third inequalities in the next type:

(24) α>ΔB×H2λminSG,H,β−2ΔA×HλminSG,H,β,α<1−1/4βΔA×c2λminSG,H,β−2ΔA×HλminSG,H,β.

And, if the next inequality will be true

(25) ΔB×H2λminSG,H,β−2ΔA×HλminSG,H,β<1−1/4βΔA×c2λminSG,H,β−2ΔA×HλminSG,H,β,

then always 0 < *α* < 1 exists, at which the second and third inequalities (23) are true. And last inequality is equivalent to the following:

(26) ΔA×H2+14βΔA×c<λminSG,H,β−2ΔA×H·λminSG,H,β.

Let us rewrite it in the type

(27) λminSG,H,β2−2ΔA×HλminSG,H,β−ΔB×H2+14βΔA×c2>0.

It will be always true, if

(28) λminSG,H,β>ΔA×H+ΔA2H2+ΔB2H2+β2ΔA2c2.

As from performance of last inequality, the performance of the first inequality (23) is similar to Theorem 3, and we obtain statement (17) of Theorem 4.

*Numerical Example*. Consider the nonlinear direct control system (5), where

(29) A=−14−12−11−20,B=−101−2,b=1−1,c=−11,

with nonlinear characteristic *f*(*σ*) located in the sector 22,5°; that is, *k* = 0.5.

As the first step, matrices *H* and *G* for the Lyapunov-Krasovskii functional (9) can be obtained as a solution of “Lyapunov matrix equation” [20]:

(30) ATH+HA=−I,BTG+GB=−I.

From Theorem 4 conditions we obtain that our system will be (Δ*A*, Δ*B*) interval absolutely stable if the next restrictions will satisfy

(31) τ≤0.01,β≤1.3,ΔA≤0.8,  ΔB≤0.8  at  β=0.2.

So, fact of system (5) stability essentially depends on its parameters (e.g., the sector solution *k*, time-delay argument *τ*) and Lyapunov-Krasovskii functional parameters (*H*, *G*, *β*). To find the “better” ones, it is possible to solve the optimization problem. But this is the other problem, and solution of it can be found, for example, at [33].

## 3. Direct Control Systems of Neutral Type

We will consider the direct control system described by the differential equations with deviating argument of neutral type and with interval given coefficients of linear part:

(32) ddtxt−Dxt−τ=A+ΔAxt+B+ΔBxt−τ+bfσt,σt=cTxt.

In system (32) we use the same notations as for system (5) from the previous part. Here is the matrix *D* that satisfies a condition of “difference operator stability”: that is, |*D*| < 1 [26]. And we need to extend initial conditions on our solution by the following [31]:

(33) $$\begin{array}{c} x\left(\;t\;\right)=\dot{\varphi }\left(\;t\;\right). \end{array}$$

In the present section for construction of absolute interval stability conditions we will use the functional of Lyapunov-Krasovskii of the following type:

(34) Vxt=xt−Dxt−τTHxt−Dxt−τ+∫−τ0xTt+sGxt+sds+β∫0σtfξdξ.

As in the previous part, we understand under definition of absolute stability the global asymptotic stability of trivial solution of the system for an arbitrary nonlinear function *f*(*σ*), which satisfies sector condition (4). We understand terms stability, asymptotic stability, and global stability of the solution of neutral type systems in the sense of definitions given in the [31].

As is known, neutral type systems have their own specific features. As a general rule, system solutions are not continuously differential function in the nodes *y* = *kτ*,  *k* = 0,1, 2,… For the obtaining of any estimates we can use the functionals of different kind. For example, it may depend or not depend on derivative of solutions [31]. Hence, we may construct some estimates of the transition process in different metrics (e.g., *C*_0_ and *C*_1_ or *L*_2_), which depends on the chosen type of functional. For Lyapunov-Krasovskii functional (34) bilateral estimates can be written as

(35) λminGxt22≤Vxt≤λmaxHxt2+D2xt−τ2+λmaxGxt22+12βkc×xt2.

That is why, in this work we formulate stability conditions of neutral type systems in the metrics ‖*x*(*t*)‖_2_ *L*_2_ space.

Let us preliminary consider the neutral system without interval perturbations:

(36) ddtxt−Dxt−τ=Axt+Bxt−τ+bfσt,σt=cTxt.

Also we will obtain absolute stability conditions of system (36).

Let us denote
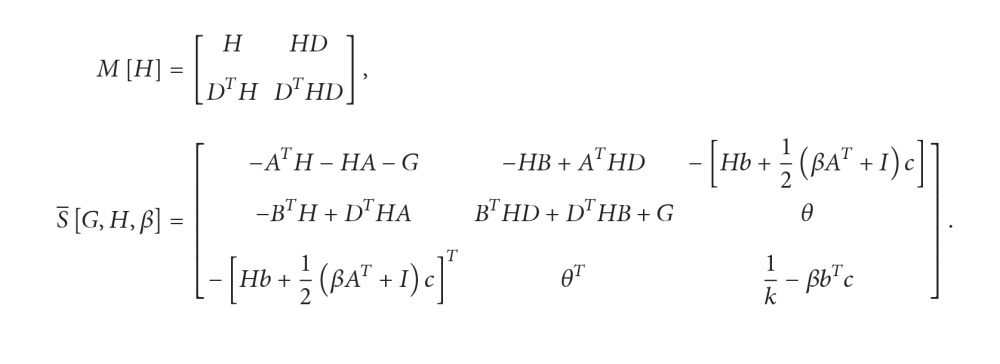
(37)

**Theorem 5 .**

Let positive definite matrices *G*, *H* exist, and parameter *β* > 0, such that the matrix $\overset{-}{S}[G,H,\beta ]$ also is positive definite. Then system (36) without interval perturbations is absolutely stable in the metrics ‖*x*(*t*)‖_2_ *L*_2_ space.

**Proof**

For Lyapunov-Krasovskii functional (34) the following bilateral estimations are true:

(38) λminGxt22≤Vxt≤λmaxMHxt2+xt−τ2+λmaxGxt22+βkσt2

or

(39) λminG×xt22Vxt≤λmaxMH+βkc2×xt2+λmaxMHxt−τ2+λmaxGxt22.

We will calculate a total derivative of functional (34) along the solutions of the system without interval perturbations. We obtain the following:

(40) ddtVxt=Axt+Bxt−τ+bfσtT·Hxt+Dxt−τ+xt−Dxt−τT·HAxt+Bxt−τ+bfσt+xTt·Gxt−xTt−τGxt−τ+βfσt·cTAxt+Bxt−τ+bfσt.

Or, using *S*-procedure [17],

(41) ddtVxt−xTt,xTt−τ,fσt·S−G,H,β·xTt,xTt−τ,fσtT,

where matrix $\overset{-}{S}\left[G,H,\beta \right]$ is defined in (37). If it is positive definite, then

(42) ddtVxt≤−λminS−G,H,β×xt2+xt−τ2+fσt2.

Thus, we have system of inequalities:

(43) λminGxt22Vxt≤λmaxMH+βkc2xt2+λmaxGxt22,ddtVxt−λminS−G,H,βxt2.

And, according to Krasovskii weak theorem [31], if there are positive definite matrices *G*, *H*, at which matrix $\overset{-}{S}\left[G,H,\beta \right]$ also is positive definite, the system is absolutely stable in the metrics ‖*x*(*t*)‖_2_ *L*_2_ space.

Further we will obtain absolute interval stability conditions of system (32).

**Theorem 6 .**

Let positive definite matrices *G*, *H* exist and parameter *β* > 0, such that the next inequality is true:

(44) $$\begin{array}{c} \lambda_{min}\left(\;\overset{-}{S}\left[\;G,H,\beta \;\right]\;\right)>\left(\;\left\|\;\Delta A\;\right\|\times \left|\;H\;\right|+\left\|\;\Delta B\;\right\|\times \left|\;H\;\right|\;\right)+\sqrt{{\left(\left\|\Delta A\right\|\times \left|H\right|+\left\|\Delta B\right\|\times \left|HD\right|\right)}^{2}+{\left(\left\|\Delta B\right\|\times \left|H\right|+\left\|\Delta A\right\|\times \left|HD\right|\right)}^{2}}. \end{array}$$

Then system (32) is (Δ*A*, Δ*B*) interval absolutely stable in the metrics ‖*x*(*t*)‖_2_ *L*_2_ space.

**Proof**

As appears from a type of functional (34), bilateral estimations (38) are true. We will calculate a total derivative of functional along solution of system with “interval perturbations.” We obtain

(45) ddtVxt−xTt,xTt−τ,fσt·S−G,H,β·xTt,xTt−τ,fσtT+xTt,xTt−τ,fσt·ΔSG,H·xTt,xTt−τ,fσtT,

where

(46) ΔSG,H=ΔATH+HΔA−HΔB+ΔAHDθ−ΔBTH+DTHΔAΔBTHD+DTHΔBθθTθT0.

If matrix $\overset{-}{S}\left[G,H,\beta \right]$ is positive definite, then

(47) ddtVxt≤−λminS−G,H,β·xt2+xt−τ2+fσt2+2ΔA×H×xt2+2ΔB×H+ΔA×HD·xt×xt−τ+ΔB×HD×xt−τ2.

From here we will have that

(48) ddtVxt≤−λminS−G,H,β−2ΔA×H×xt2+2ΔB×H+ΔA×HD×xt×xt−τ−λminS−G,H,β−2ΔB×HD×xt−τ2−λminS−G,H,βfσt2.

Then, as appears from Sylvester's criterion [32], performance of system of inequalities will be a condition of absolute interval stability:

(49) λminS−G,H,β−2ΔA×H>0,λminS−G,H,β−2ΔA×H×λminS−G,H,β−2ΔB×HD−ΔB×H+ΔA×HD2>0.

Let us rewrite the second inequality in a type:

(50) λminS−G,H,β2−2ΔA×H+ΔB×HDλminS−G,H,β−ΔB×H−ΔA×HD2>0.

It will be true especially if there will be positive definite matrices *G*, *H* and parameter *β* > 0, such that

(51) $$\begin{array}{c} \lambda_{min}\left(\;\overset{-}{S}\left[\;G,H,\beta \;\right]\;\right)>\left[\;\left\|\;\Delta A\;\right\|\times \left|\;H\;\right|+\left\|\;\Delta B\;\right\|\times \left|\;HD\;\right|\;\right]+\sqrt{{\left[\left\|\Delta A\right\|\times \left|H\right|+\left\|\Delta B\right\|\times \left|HD\right|\right]}^{2}+{\left[\left\|\Delta B\right\|\times \left|H\right|+\left\|\Delta A\right\|\times \left|HD\right|\right]}^{2}}. \end{array}$$

From here the statement of Theorem 6 follows.

Directly from Theorem 6 the consequence, which is easier realized by checking out of the conditions of interval stability, follows.

*Consequence*. Let positive definite matrices *G*, *H* exist and parameter *β* > 0, such that the inequality is true:

(52) λminS−G,H,βλmaxH>ΔA+ΔB+ΔA+ΔB×D2+ΔB+ΔA×D2.

Then system (32) is (Δ*A*, Δ*B*) interval absolute stable in the metrics ‖*x*(*t*)‖_2_ *L*_2_ space.

## 4. Conclusion and Prospects

In the paper, the nonlinear systems of automatic control described in terms of the ordinary differential equations with delay and neutral type, and also having uncertainties in the set of linear parts, are received constructive algebraic criteria of interval absolute stability. At the expense of application of the alternative approach of Lyapunov-Krasovskii functional, forms of estimations in sufficient conditions of interval stability are essentially simplified in comparison with obtained analogous one on the basis of finite-dimensional Lyapunov's functions of Lur'e-Postnikov types [24, 25, 34, 35].

In the chosen approach results can be extended further on; the so-called critical case (indirect control system) is perspective. Besides, applying the specified approach, similar results for the discrete systems are obtained [36, 37]. It study is actually enough recently. Also from the point of view of authors interest in the future represents construction of Lyapunov functions and Lyapunov-Krasovskii functionals, which are optimal in the classes by the set criteria of quality, for example, [33].

The next fact also should be noted: if the conditions of Theorems 3–6 failed to satisfy, it is not a dead-end situation. In such case, you can go, for example, to the solving of the stabilization problem to a state of absolute stability [38, 39].

All this confirms the viability and prospects of Lyapunov's direct method in the qualitative analysis of complex dynamical systems.
